# The ımpact of demographic and obstetric factors on perception of traumatic birth and breastfeeding attitudes

**DOI:** 10.61622/rbgo/2025rbgo15

**Published:** 2025-04-30

**Authors:** Ayşe Çuvadar, Elnaz Karamelikli, Yeter Çuvadar Baş

**Affiliations:** 1 Karabük University Faculty of Health Sciences Department of Midwifery Karabük Turkiye Department of Midwifery, Faculty of Health Sciences, Karabük University, Karabük, Turkiye.; 2 Gedik University Vacational School İstanbul Türkiye Vacational School, Gedik University, İstanbul, Türkiye.

**Keywords:** Breast feeding, Delivery, obstetric, Parturition, Postpartum period, Sociodemographic factors, Perception, Stress disorders, post-traumatic

## Abstract

**Objective:**

This study aims to examine the effects of sociodemographic and obstetric factors on traumatic birth perception and breastfeeding attitudes in primiparous mothers who have had a vaginal birth in the early postpartum period.

**Methods:**

The sample of the research, developed with a cross-sectional and correlational design, consisted of 252 women residing in a province in the Western Black Sea region of Türkiye. The data were obtained by employing a Personal Information Form, Traumatic Childbirth Perception Scale, and Breastfeeding Attitudes of The Evaluation Scale. Data analysis was conducted using the statistical programming language R (R version 4.3.3).

**Results:**

Women who were not employed, had a planned pregnancy, and did not experience health problems during pregnancy had higher mean breastfeeding attitude scores, and this difference was statistically significant. It was determined that a one-unit increase in gestational week led to an average increase of 1.926 units in breastfeeding attitude score, and a one-unit increase in Traumatic Childbirth Perception Scale score led to an average decrease of 0.110 units in breastfeeding attitude score. The mean traumatic childbirth perception scores of women living in urban areas were found to be lower than those living in villages or towns, and the difference was statistically significant.

**Conclusion:**

The research findings indicate that gestational age, perception of traumatic childbirth, and certain sociodemographic factors significantly affect breastfeeding attitudes. Additionally, mothers living in urban areas have a lower perception of traumatic childbirth. Therefore, individualized approaches to childbirth and breastfeeding support are crucial.

## Introduction

Traumatic birth experience can be defined as when a woman feels at any stage of the birth process that in situations where there is a substantial risk of serious harm or fatality to the mother or her infant.^(1)^ The perception of traumatic birth is shaped by women's sociodemographic characteristics (education level, pregnancy planning status, etc.),^(1)^ obstetric and gynecological features (mode of delivery, number of births, difficult or interventionist delivery, history of traumatic birth) and is also influenced by factors such as routine practices during childbirth (episiotomy, operative vaginal delivery, etc.).^(2)^ A birth experience marked by trauma may lead to can have negative effects on women's health in the short and/or long term. According to research, one third of women experience the birth process as a traumatic experience.^(3,4)^ Perceiving birth as traumatic can lead to the emergence indicators of post-traumatic stress in women in the postpartum period, as well as psychological problems such as the development of post-traumatic stress or depressive symptoms.^(5)^ Additionally, a traumatic birth experience can lead to decreased breastfeeding self-efficacy, difficulty bonding with the baby, a preference for cesarean delivery in subsequent births, and neglect of the baby.^(6-9)^

In addition to adversely affecting maternal mental health, a traumatic birth experience negatively impacts breastfeeding attitudes.^(10,11)^ Studies have reported that women who experience traumatic births feel detached during breastfeeding, show minimal interest in their babies, and rarely make eye contact or interact with them.^(10)^ Issues arising during labor, medical interventions, inadequate support from healthcare professionals, and a heightened perception of birth trauma are associated with negative breastfeeding attitudes.^(10,11)^

According to WHO guidelines, infants are recommended to consume only breast milk during their initial six months; starting from the 6th month, it is advised to continue breastfeeding along with adequate and safe complementary foods (solid and liquid) until the age of 2.^(12)^ Breastfeeding strengthens the bond between mother and baby and contributes to the development of both the physical and mental health of society.^(10,13)^ Health professionals can support mothers to breastfeed during pregnancy and can change mothers’ behaviors during the breastfeeding period. However, as it is known, behavior is influenced not only by knowledge but also by a woman's attitude.^(14)^ In his study, Yildiz (2019)^(15)^ states that demographic and obstetric factors including variables such as age, level of education income bracket and number of births play an important role in breastfeeding success and attitudes of mothers.

The childbirth process is a critical period in a woman's life, both physically and psychologically. Traumatic experiences during this time can negatively impact the mother's mental health, breastfeeding attitudes, and the bond she forms with her baby. Breastfeeding is vital for the healthy development of infants and the postpartum well-being of mothers.^(16)^ Previous studies in the literature indicate that traumatic birth perception and breastfeeding self-efficacy are influenced by demographic and obstetric factors, but studies evaluating breastfeeding attitudes are quite limited in numbe.^(7)^ This study aims to fill this gap by analyzing how sociodemographic and obstetric factors influence both traumatic birth perception and breastfeeding attitudes among primiparous mothers in the early postpartum period. Understanding these relationships is essential for healthcare professionals to develop individualized support strategies during childbirth and breastfeeding, ultimately enhancing maternal and infant health outcomes.

### Questions of the research

1. How do sociodemographic factors (age, education level, employment status, place of residence) affect mothers’ breastfeeding attitudes and perceptions of traumatic birth?
2. How do obstetric factors (pregnancy planning status, health issues during pregnancy, gestational age) influence mothers’ breastfeeding attitudes and perceptions of traumatic birth?
3. What is the relationship between mothers’ perceptions of traumatic birth and their breastfeeding attitudes?

## Methods

The design of this study aimed to as a cross-sectional and relationship-seeking research to evaluate the relationships between the demographic and obstetric characteristics, birth experiences, and breastfeeding perspectives of primiparous women who delivered vaginally in Karabük from 15 November 2023 to 15 July 2024.

The sample of the research consisted of 252 women residing in a province situated in Turkey's Western Black Sea region. Data was collected from women meeting the inclusion criteria, reached through an online survey using the snowball sampling method. In this method, initial participants facilitated access to other potential participants, thereby expanding the sample through referrals from women meeting the research criteria.

*Inclusion criteria:*

- Being over 18 years old;
- Undergoing vaginal birth;
- Being a primiparous participant;
- Willingly joining the study;
- Having internet access and the capability to complete the data collection form sent through WhatsApp.

*Exclusion criteria:*

- Having a chronic illness;
- Participating in the study but leaving the surveys and scales unfinished;
- Having received a diagnosis of a psychiatric illness.

The data collection process was conducted between November 15, 2023, and July 15, 2024. Initially, acquaintances and healthcare professionals who met the research criteria were contacted to reach participants. Through these individuals, other potential participants were approached, and an online survey link was shared via WhatsApp. Participants completed the survey at their convenience in their own time and preferred locations. Throughout the data collection process, participants’ confidentiality and anonymity were maintained.

In this study, the Personal Information Form, Traumatic Childbirth Perception Scale, and Breastfeeding Attitudes Evaluation Scale^(17,18)^ were used for data collection. Permission has been obtained from the authors for the Traumatic Childbirth Perception Scale and the Breastfeeding Attitudes Evaluation Scale. The data collection tools of the study were distributed through a invitation sent to participants via WhatsApp and Instagram platforms, as well as a survey link that could be filled out on a secure website. Before taking part in the study, participants were requested to complete an informed consent form. The collected data was automatically transferred to an Excel file.

A personal information form was developed by the researchers, drawing on existing literature, to assess the mothers’ demographic profiles (age, education level, occupation, income level, etc.) and obstetric characteristics (number of pregnancies, method of delivery, complications during childbirth, etc.).

This scale was created by Yalniz et al.^(17)^ to assess women's perceptions of traumatic birth. The scale consists of 13 items, each scored between 0 (positive view) and 10 (negative view). The total score ranges from 0 to 130, determining the level of traumatic birth perception from low to high. In this context, the range of 0-26 points is considered "very low," 27-52 points is considered "low," 53-78 points is considered "moderate," 79-104 points is considered "high," and 105-130 points is considered "very high" traumatic birth perception. The internal consistency of the scale was found to be 0.895 with Cronbach's alpha coefficient. The reliability analysis yielded a Cronbach's alpha coefficient of 0.908 for this study.

The Scale was created by Arslan^(18)^ to evaluate the breastfeeding attitudes of mothers who have given birth. The Scale is in a 5-point Likert type and is scored between 0 and 4. The Scale consists of 46 questions, including 22 positive and 24 negative breastfeeding attitude items. The scale's total score spans from 0 up to 184 points. As the score obtained from the Scale increases, mothers’ attitudes towards breastfeeding are considered positive. The internal consistency of the scale, as measured by Cronbach's alpha, is reported to be 0.63.^(18)^ In this study, the Crombach's alpha coefficient was found to be 0.62. In the literature, a Cromba alpha value between 0.60-0.79 is considered to indicate that the test is quite reliable.^(19)^

Data analysis was performed using the statistical programming language R (R version 4.3.3). Statistical analyses were conducted with the following steps; Frequency, percentage, mean, and standard deviation (X ± SD) values were calculated for descriptive statistics. The internal consistency of the scales used in this study was assessed by calculating Cronbach's α coefficient. One-way analysis of variance (ANOVA) and independent samples t-test were used in intergroup comparisons. In ANOVA tests, post-hoc tests (Tukey HSD) were conducted for detailed examination between groups where significance was obtained. The relationships between mothers’ age, length of marriage, birth week, traumatic birth perception, and breastfeeding attitude were examined using multiple linear regression analysis. The explanatory level of the model was evaluated with the coefficients of R-squared (R²) and adjusted R-squared (adjR²). The analysis includes reporting of standard errors, confidence intervals (CI), t-values, and p-values. Pearson correlation analysis was used to determine the relationships between mothers’ age, length of marriage, birth week, traumatic birth perceptions, and breastfeeding attitudes. Correlation coefficients (r) and significance levels of relationships (p) have been reported. A significance threshold of p<0.05 was applied to all statistical tests. The results were interpreted with a 95% (CI. Missing data were managed during the analysis with appropriate functions in R.

The research process was performed in alignment with the principles of the Helsinki Declaration on Human Rights. The Non-Interventional Clinical Research Ethics Committee of a university granted ethical approval for this study (Decision No: 2023/1451, Date: 10.11.2023). Written consent has been obtained from women before the study.

## Results

When the socio-demographic characteristics of mothers were compared with the average scores of BAES and TCPS; The analysis indicated no significant statistical discrepancy was observed in the mean scores of BAES and TCPS concerning education level, income status, pregnancy-related education, birth complications, or the use of forceps/vacuum interventions. Mothers who are housewives have been found to have higher BAES scores compared to working mothers, and there is a notable statistical difference between the groups. However, there appears to be no meaningful statistical difference in TCPS scores between them. Mothers living in the village have higher TCPS scores compared to mothers living in the city and district, and the difference between them is statistically significant. However, there is no significant difference in BAES score averages. Mothers with planned pregnancies have higher BAES scores on average, and the difference between them is significant, but there is no significant difference in perceived traumatic birth experiences scores. Mothers experiencing health problems during pregnancy have higher BAES scores compared to those who do not, and mothers who undergo episiotomy during birth have higher BAES scores than those who do not; however, TCPS scores remain statistically unchanged (Table 1).

**Table 1:** Evaluation of the relationships between demographic, obstetric, and birth experience characteristics of mothers and their perceptions of traumatic birth and breastfeeding attitudes

Variebles		n(%)	BAES		TCPS	
			Mean ± SD	Test statistics /p	Mean ± SD	Test statistics /p
Employment status						
	Housewife	175(69.44)	108.5±1	F=3.262247	74.7±1.96	F=0.5480823
	Worker	25(9.92)	99.8±2.49	p=0.022	77.2±5.14	p=0.649
	Employee	37(14.68)	106.7±2.06		69.6±4.17	
	Her own business	15(5.95)	106.1±3.9		75.9±6.23	
Educational status						
	Elementary School	24(9.52)	107.9±2.59	F=1.672704	75.7±5.58	F=1.217025
	Middle School	37(14.68)	102.9±2.31	p=0.173	81.4±3.93	p=0.304
	High School	94(37.3)	107.4±1.41		72.5±2.7	
	University	97(38.49)	108.6±1.3		73±2.57	
Where do you live?						
	Village	37(14.68)	108.2±2.33	F=0.3116307	87.8±4.34	F=7.055603
	Town	93(36.9)	106.4±1.37	p=0.732	74.3±2.35	p=0.001
	City	122(48.41)	107.6±1.2		70.2±2.39	
İncome status						
	Income equals expenses	155(61.51)	108.5±1.11	F=2.275816	74.3±2.04	F=0.0753935
	Income is more than expenses	23(9.13)	107.2±.54	p=0.104	72.5±4.35	p=0.927
	Income is less than expenses	74(29.37)	104.5±1.43		74.9±3.23	
Was the pregnancy planned?						
	Yes	172(68.25)	109.5±0.99	t=4.166271	72.8±1.98	t=-1.372193
	No	80(31.75)	102.3±1.44	p<0.001	77.5±2.79	p=0.171
Did you receive education related to pregnancy?						
	Yes	111(44.05)	108.6±1.18	t=1.473006	70.9±2.38	t=-1.877618
	No	141(55.95)	106.1±1.18	p=0.142	77±2.18	p=0.061
Did you experience any health problems during pregnancy?						
	Yes	54(21.43)	103.3±1.75	t=-2.509211	72.2±3.87	t=-0.6225076
	No	198(78.57)	108.3±0.95	p=0.013	74.9±1.77	p=0.535
Did you experience any complications during birth?						
	Yes	16(6.35)	102.2±3.12	t=-1.663027	77.3±7.74	t=0.3998132
	No	236(93.65)	107.6±0.87	p=0.114	74.1±1.65	p=0.694
Was vacuum/forceps intervention applied at birth?						
	Yes	7(2.78)	102.6±5.33	t=-0.8875704	83±9.71	t=0.9097344
	No	245(97.22)	107.4±0.85	p=0.407	74±1.64	p=0.396
Was an episiotomy performed during birth?						
	Yes	153(60.71)	105.4±1.11	t=-2.7877	76.4±1.91	t=1.54241
	No	99(39.29)	110±1.24	p=0.005	71.1±2.85	p=0.124

SD: Standard deviation, BAES: Breastfeeding Attitudes Evaluation Scale, TCPS: Traumatic Childbirth Perception Scale

To estimate the effects of participants’ age, marriage length, gestational week, and TCPS scores on BAES, a multiple linear regression analysis was applied. According to the analysis results, the model created was found to be statistically significant (F=5.411, p < 0.001). The model indicates that 39.2% of the variance in the dependent variable is explained by the independent variables (R = 12.9, R² = 0.08057, Adjusted R2 = 0.06568). Among the independent variables included in the model, gestational week and TCPS were determined as significant predictors of breastfeeding attitude (p < 0.001). The constant coefficient was found to be significant (B = 39.203, t = 1.446, p = 0.149), indicating that when the effects of all other variables are zero, the average of the dependent variable is 39.203. The gestational week coefficient was found to be significant and positively correlated (B = 1.926, t = 2.910, p = 0.003), indicating that a one-unit increase in the gestational week resulted in an average increase of 1.926 units in breastfeeding attitude score. The TCPS coefficient was found to be significant and negative (B = -0.110, t = -3.449, p < 0.001), indicating that a one unit increase in TCPS score leads to an average decrease of 0.110 units in breastfeeding attitude score. Age and length of marriage factors are not statistically significant predictors (Table 2).

**Table 2:** Factors influencing the breastfeeding attitudes evaluation scale

İndependent variable	Unstandardized coefficients B	Standardized coefficients		T	p-value	95.0% CI
		SE	β			
Constant	39.203	27.107		1.446	0.149	-13.925 to 92.333
Age	0.016	0.211	0.006	0.076	0.939	-0.398 to 0.430
Length of marriage	0.175	0.237	0.061	0.739	0.460	-0.289 to 0.640
Gestational week	1.926	0.661	0.180	2.910	0.003	0.629 to 3.224
TCPS	-0.110	0.032	-0.212	-3.449	<0.001	-0.173 to -0.047

F= 5.411, p<0.001; R:12.9; R^2^=0.08057; Adjusted R^2^=0.06568; CI, confidence interval; SE, standard error; β,standardized regression coefficient, TCPS; Traumatic Childbirth Perception Scale;

This model examined the effects of age, length of marriage, gestational week, and BAES scores on TCPS. According to the analysis results, the model was found to be statistically significant (F=4.213, p=0.002), it is observed that the model only explains 6.3% of the variance in the dependent variable (R = 25.05, R² = 0.06387, Adjusted R² = 0.04871). BAES has been identified as a significant predictor of TCPS. Length of marriage, age, and gestational week factors are not statistically significant predictors of traumatic birth perception. The coefficient of B = -0.416 in BAES was found to be significant and in the negative direction (p < 0.001). This indicates that a one unit increase in BAES score results in an average decrease of 0.416 units in TCPS score (Table 3).

**Table 3:** Factors influencing the traumatic childbirth perception scale

İndependent variable	Unstandardized coefficients	Standardized coefficients		T	p-value	95.0% CI
	B	SE	β			
Constant	10.070	52.447		1.927	0.055	-1.726 to 203.866
Age	-0.793	0.407	-0.162	-1.948	0.052	-1.592 to 0.004
Length of marriage	0.895	0.457	0.163	1.956	0.051	-0.001 to 1.791
Gestational week	0.905	1.305	0.044	0.693	0.488	-1.653 to 3.463
BAES	-0.416	0.120	-0.216	-3.449	<0.001	-0.652 to -0.179

F= 4.213, p<0.001; R=25.05; R^2^=0.06387; Adjusted R^2^=0.04871 Abbreviations: CI, confidence interval; SE, standard error; β,standardized regression coefficient, BAES; Breastfeeding Attitudes Evaluation Scale;

A weak positive correlation was found between birth week and breastfeeding attitude, and this relationship was statistically significant (r=0.179, p=0.004). A statistically significant but weak negative correlation was observed between the perception of traumatic birth and breastfeeding attitude (r=-0.204, p=0.001) (Schober et al.)^(20)^ (Table 4).

**Table 4:** The relationship between mothers’ age, length of marriage, and birth week with BAES

		Age	Length of marriage	Gestational week	TCPS
BAES	r	0.048413	0.0716008	0.179065	-0.204577
	p	0.0441761	0.2574534	0.0043511	0.0010907

r: Pearson Correlation, BAES: Breastfeeding Attitudes Evaluation Scale, TCPS: Traumatic Childbirth Perception Scale

**Figure 1:**
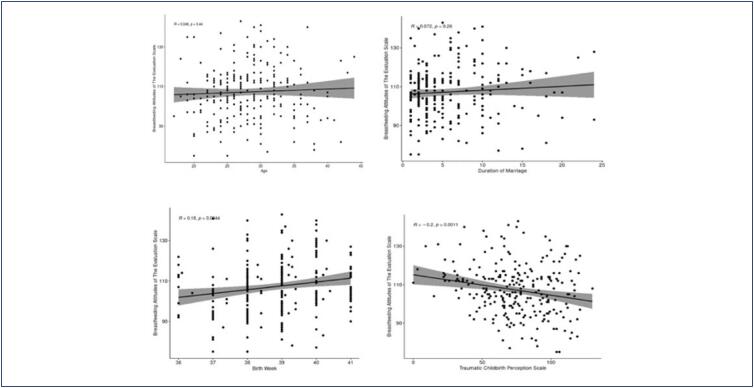
The relationship graph between mothers’ breastfeeding attitudes and age, length of marriage, and perception of traumatic birth

**Figure 2:**
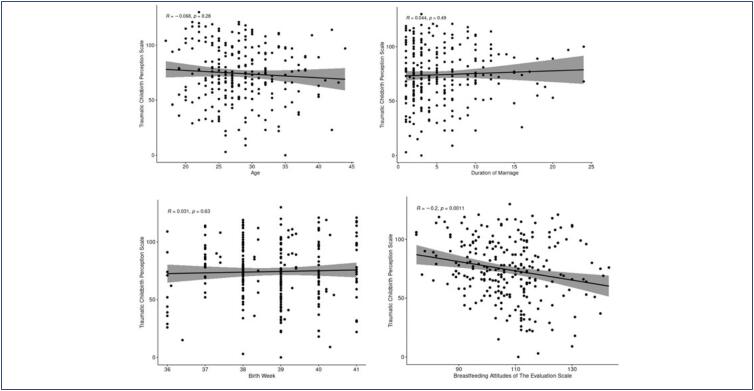
The relationship graph between mothers’ traumatic birth perception and age, length of marriage, and breastfeeding attitude

There is no statistically significant relationship between traumatic birth perception and age, length of marriage, and week of birth (Table 5) (Figures 1 and 2).

**Table 5:** The relationship between mothers’ age, length of marriage, and birth week with TCPS

		Age	Length of marriage	Gestational week	BAES
TCPS	r	-0.0682333	0.0440839	0.030907	-0.204577
	p	0.8597158	0.2430049	0.3126648	0.0010907

r: Pearson Correlation, BAES: Breastfeeding Attitudes Evaluation Scale, TCPS: Traumatic Childbirth Perception Scale

## Discussion

Within the scope of this research, where the goal was to compare the effects of mothers’ socio-demographic and obstetric characteristics on BAES scores and TCPS scores, birth week and TCPS score were found to be statistically significant predictors of breastfeeding attitude. The study's findings indicate that gestational age, perception of traumatic birth, and certain sociodemographic factors significantly influence breastfeeding attitudes. Additionally, mothers residing in urban areas have a lower perception of traumatic birth.

No statistically significant variation was found between the average scores of BAES and TCPS in terms of women's education, income status, receiving education related to pregnancy, experiencing complications during childbirth, and undergoing forceps/vacuum interventions during childbirth. Studies in the literature support that education and receiving education during pregnancy are not associated with breastfeeding attitudes.^(21)^ In another study, it has been reported that holding a higher educational background and a positive interpretation towards breastfeeding not only motivates mothers to breastfeed more, but also leads them to have confidence in breastfeeding.^(22)^ The lack of a significant difference between income level and breastfeeding attitude contradicts the findings of Mohammed and Soliman,^(23)^ which showed that mothers with higher income levels have more positive attitudes towards breastfeeding. This situation can be explained by the differentiation of cultural and economic factors that arise in different socio-economic situations. Study of Corby et al.^(24)^ study suggested that pregnancy education can contribute to the breastfeeding process. Factors such as education, social support, and awareness level can be considered important in this interaction.

There is a difference between stay-at-home mothers and working mothers in terms of breastfeeding attitudes, which is inconsistent with the finding of Ghasemi et al.^(21)^ that breastfeeding attitudes are shaped more by self-confidence and knowledge level than socio-demographic factors. This discrepancy may be attributed to the studies being conducted in different geographical regions.

The higher TCPS scores of mothers living in rural areas compared to those living in urban areas are related to difficulties in accessing healthcare services and lack of social support.^(25)^ Inadequate healthcare services and traditional perceptions of childbirth in rural areas may cause mothers to perceive childbirth as a more traumatic experience.^(26)^ A study has indicated that negative experiences with healthcare workers in rural areas increase the perception of trauma. Another study also shows that psychological support plays a critical role in reducing this perception.^(27)^ In the study by Bohren et al.,^(28)^ it is stated that inadequate health services and complications during childbirth lead mothers to perceive birth as more traumatic. In rural areas, when giving birth, the lack of adequate services in situations requiring emergency medical intervention can turn childbirth into a traumatic experience. According to another study, postnatal mental health support plays a critical role in reducing mothers’ perception of traumatic birth. The lack of access to such support services in rural areas can negatively affect mothers’ ability to cope with trauma after birth and lead to a lasting perception of traumatic birth.^(29)^

In this study, BAES scores did not vary between mothers from rural and urban settings. Contrarily, a study with urban Malaysian mothers reported that family involvement is key in promoting supportive breastfeeding attitudes.^(30)^

The higher BAES scores of mothers experiencing planned pregnancies are similar to the literature.^(21,25)^

Despite the findings in the literature that experiencing health problems during pregnancy, undergoing episiotomy, and facing complications during childbirth increase the perception of traumatic birth,^(25,26)^ this study found that these factors did not lead to a considerable effect on the perception of traumatic childbirth in the TCPS. These results indicate that the perception of traumatic birth should be addressed not only in terms of physical complications but also within a broader framework including the individual's psychosocial status, level of preparedness for childbirth, and support systems. Although no significant effect on the perception of traumatic birth was determined, experiencing complications during pregnancy and undergoing an episiotomy during childbirth negatively impacted women's breastfeeding attitudes. This finding aligns with the literature.^(10,31)^ This result is also consistent with studies indicating that fear and stress experienced by women during childbirth can adversely affect the lactation process.

The fact that gestational age is a statistically significant predictor of breastfeeding attitudes suggests that as pregnancy progresses, mothers develop more positive attitudes toward breastfeeding. This finding aligns with Ghasemi et al.^(21)^ This may be explained by the increased knowledge, confidence, and preparedness for breastfeeding that mothers acquire as they approach their due date. Additionally, the strengthening of the maternal-fetal bond and heightened awareness of the importance of breastfeeding in the later stages of pregnancy may positively influence breastfeeding attitudes.

Traumatic birth perception has been found to be a statistically significant predictor of breastfeeding attitude. It has been observed that mothers who have experienced traumatic birth have lower breastfeeding motivation. Studies in the literature indicate that traumatic birth perception reduces breastfeeding attitude.^(32)^Another study reported that women who went through traumatic births faced challenges with breastfeeding.^(33)^ In a qualitative study investigating the breastfeeding experiences of mothers after birth trauma, it was reported that women who experienced birth trauma avoided breastfeeding their babies.^(34)^ This relationship shows that the perception of experiencing a traumatic birth may lead to lasting effect on both the health of mothers and babies. In this study, it was observed that breastfeeding attitude negatively predicted the perception of traumatic birth and that the gestational week had a positive effect on this relationship.

Age and duration of marriage are not statistically significant predictors of breastfeeding attitude. This finding indicates that these demographic factors do not directly affect breastfeeding attitudes. It has also been reported that the effects of socio-demographic factors on breastfeeding behaviors in a study may be more complex and that cultural factors may also play a role in this interaction.^(21)^ In another study, it was stated that the age of mothers and the duration of marriage did not have a direct impact on breastfeeding attitudes.^(23)^ It has been emphasized in another study that breastfeeding attitudes are more related to the mother's psychosocial status rather than demographic variables such as age and duration of marriage.^(35)^

There are some limitations to this research. First, since the data were collected by using the online survey method, the possibility of bias cannot be ignored. Second, the fact that all mothers are from the province of Karabük may limit the generalizability of these results to other regions. It is recommended to carry out additional studies covering wider regions. In the literature, there are studies indicating that traumatic birth perception and breastfeeding self-efficacy are influenced by demographic and obstetric factors, but studies evaluating breastfeeding attitudes are quite limited in number.

The findings of this study provide valuable contributions to the midwifery literature by elucidating the impact of mothers’ sociodemographic and obstetric characteristics, as well as their traumatic birth experiences, on breastfeeding attitudes.

Firstly, it was observed that mothers who give birth at earlier gestational weeks have lower breastfeeding attitude scores. This indicates the necessity of developing special breastfeeding support programs for mothers who have preterm deliveries. These programs are believed to increase mothers’ motivation for breastfeeding by providing psychosocial support both before and after birth. Additionally, it is crucial to provide psychosocial support and guidance services to mothers with a high perception of birth trauma, considering the negative impact on their breastfeeding attitudes. These interventions can help reduce the psychological effects of birth trauma and enable mothers to approach the breastfeeding process more positively. Therefore, healthcare professionals, especially midwives and nurses, should be trained to offer more support to mothers who have experienced traumatic births.

## Conclusion

In conclusion, this study reveals that the impact of mothers’ socio-demographic and obstetric characteristics on breastfeeding attitudes and perceptions of traumatic birth is complex and multifaceted. Factors such as education, income level, and employment status do not have a significant impact on breastfeeding attitudes, however, a one-unit increase in the week of gestational results in an average increase of 1.926 units in breastfeeding attitude scores. Additionally, evidence suggests that perceiving a birth as traumatic negatively influences breastfeeding attitudes, and this relationship can affect both the physical and psychological well-being of mothers. Considering that the results show that mothers who give birth at a later gestational age have more positive breastfeeding attitudes, it may be beneficial to develop special breastfeeding support programs for mothers who give birth prematurely. Additionally, it is important to provide psychosocial support and guidance services aimed at reducing the perception of traumatic birth in mothers with high TCPS scores in order to improve their breastfeeding attitudes. Healthcare professionals should provide individualized support during childbirth and breastfeeding processes by considering mothers’ sociodemographic and obstetric characteristics and implement interventions aimed at reducing the perception of traumatic birth. This approach can positively influence breastfeeding attitudes and support maternal and infant health.
